# Staged stromal extracellular 3D matrices differentially regulate breast cancer cell responses through PI3K and beta1-integrins

**DOI:** 10.1186/1471-2407-9-94

**Published:** 2009-03-26

**Authors:** Remedios Castelló-Cros, David R Khan, Jeffrey Simons, Matthildi Valianou, Edna Cukierman

**Affiliations:** 1 Cancer Genetics and Signaling Program, Fox Chase Cancer Center, Philadelphia, PA 19111-2497, USA

## Abstract

**Background:**

Interactions between cancer cells and stroma are critical for growth and invasiveness of epithelial tumors. The biochemical mechanisms behind tumor-stromal interactions leading to increased invasiveness and metastasis are mostly unknown. The goal of this study was to analyze the direct effects of staged stroma-derived extracellular matrices on breast cancer cell behavior.

**Methods:**

Early and late three-dimensional matrices were produced by NIH-3T3 and tumor-associated murine fibroblasts, respectively. After removing fibroblasts, extracted matrices were re-cultured with breast epithelial cells of assorted characteristics: MCF-10A (non-tumorigenic), MCF-7 (tumorigenic, non-invasive), and MDA-MB-231 (tumorigenic, invasive). Effects prompted by staged matrices on epithelial cell's growth, morphology and invasion were determined. Also, matrix-induced velocity, directionality and relative track orientation of invasive cells were assessed in the presence or absence of inhibitors of phosphoinositide-3 kinase (PI3K) and/or beta-1 integrin.

**Results:**

We observed that assorted breast epithelial cells reacted differently to two-dimensional vs. staged, control (early) and tumor-associated (late), three-dimensional matrices. MCF-10A had a proliferative advantage on two-dimensional substrates while MCF-7 and MDA-MB-231 showed no difference. MCF-10A and MCF-7 formed morphologically distinguishable aggregates within three-dimensional matrices, while MDA-MB-231 exhibited increased spindle-shape morphologies and directional movements within three-dimensional matrices. Furthermore, MDA-MB-231 acquired a pattern of parallel oriented organization within tumor-associated, but not control matrices. Moreover, tumor-associated matrices induced PI3K and beta1-integrin dependent Akt/PKB activity in MDA-MB-231 cells. Interestingly, beta1-integrin (but not PI3K) regulated tumor-associated matrix-induced mesenchymal invasion which, when inhibited, resulted in a change of invasive strategy rather than impeding invasion altogether.

**Conclusion:**

We propose that both cells and matrices are important to promote effective breast cancer cell invasion through three-dimensional matrices and that beta1-integrin inhibition is not necessarily sufficient to block tumor-matrix induced breast cancer cell invasion. Additionally, we believe that characterizing stroma staging (e.g., early vs. late or tumor-associated) might be beneficial for predicting matrix-induced cancer cell responses in order to facilitate the selection of therapies.

## Background

Metastasis, as opposed to tumor growth, is the major cause of cancer mortality, accounting for 90% of deaths in solid neoplasias [1], such as breast cancer. Furthermore, the American Cancer Society has identified breast cancer as the number one neoplasia in women in the United States [2]. It is well established that both transformed epithelial cells and their associated stromal microenvironment are active contributors to the development of mammary and other epithelial cancers [3-5], and that stromal paracrine effects induce epithelial cell tumorigenic responses [3], such as increased proliferation [4,6] and metastasis [7-10]. In breast carcinomas, changes in the stroma include appearance of discontinuities in the basement membrane surrounding the growing tumor, immune responses, formation of new vessels, and a desmoplastic reaction that includes activated fibroblasts (myofibroblasts) and remodeling of their mesenchymal extracellular matrix (ECM) [11-15]. In addition, both direct and indirect interactions between cancer cells and the mesenchyme are responsible for triggering the activation of the tumor-associated stroma (e.g., desmoplasia), creating a permissive environment in support of tumor development and cell invasion [5,13,16].

Plasticity of tumor-associated stroma consists of both molecular and topographical changes that result in part from altered amounts and availability of matrix-modification proteins such as proteases [17], which contribute to variations in organization and pliability (e.g., stiffness) of the ECM [18,19]. As a result of these types of tumor-induced stromal modifications, the microenvironment differentially engages cell-matrix receptors like the integrins, which in turn alter cell responses such as cancer cell invasion [20-22]. Moreover, topographical reorganization of the ECM, such as the presence of parallel oriented patterns of collagen fibers, facilitates local cell invasion [15]. Regarding types of invasive strategies, investigators have proposed that single cell invasion could occur by either epithelial-to-mesenchymal transitioned movement or by an amoeboid-like strategy, and that collective cell invasion could involve micro- or macro-track cell formations, all of which depend on microenvironmental characteristics [15,23-25]. Interestingly, it has also been proposed that tumor cells can transition between these invasive strategies in response to tumor-induced stromal plasticity [26,27].

Integrins, which are trans-membrane adhesive receptors that are composed of heterodimeric subunits designated as alpha and beta, are responsible for perceiving and responding to changes in both the extracellular microenvironment and the inner cell by linking the ECM to the cytoskeleton [28]. It has been suggested that beta1-integrins, which represent the largest integrin subfamily, play a central role in tumor cell responses that include invasion and metastasis [29-31]. For example, beta-1 integrins, among others, have been implicated in the regulation of protein Kinase B (PKB) also known as Akt [32,33], which consecutively plays crucial roles in regulating breast cancer cell invasion [34-36]. Furthermore, inhibition of beta-1 integrin has been shown to result in a change of invasive strategy [37,38].

Due to the influence of the mesenchymal microenvironment on cancer invasion [39], much effort has been invested in developing 3D model systems that effectively mimic the *in vivo *microenvironmental settings [40-42]. Since little is known about the direct effects that tumor-associated mesenchymal ECMs have on breast epithelial cell responses during tumor invasion, we have developed an *in vivo*-like 3D ECM system [43]. In fact, the original system has recently been modified to allow the use of a variety of fibroblasts, which produce self-derived 3D matrices that mimic successive stages of tumor-induced stroma progression [44,45]. For instance, 3D ECMs derived from NIH-3T3 fibroblasts resemble matrices obtained from primary fibroblasts isolated from primed or pre-disposed tumor-associated stroma, and hence are regarded as control or early 3D ECMs [44,46]. Similarly, 3D ECMs obtained from primary fibroblasts harvested from tumor samples resemble late *in vivo *or activated (e.g., desmoplastic) stromal matrices, which present tumor-associated stromal characteristics such as the above-mentioned topographical parallel-organized patterns of ECM fibers [44,46]. We believe that staged ECMs can be used as 3D substrates for epithelial cells in order to study tumor-associated ECM induced responses such as growth, cell morphology, and cell invasion [46-48]. Consequently, in the first part of this study and as proof of principle, we tested the direct effects that *in vivo*-like control and tumor-associated mesenchymal 3D ECMs have on immortalized normal (MCF-10A), tumorigenic (estrogen receptor modified MCF-7, [49]) and metastatic (MDA-MB-231) breast epithelial cells. Furthermore, we investigated the influences imparted by early (control) vs. late (tumor-associated) staged 3D ECMs on the regulation of both the morphological features and the invasive strategies of MDA-MB-231 cells through engagement of beta1-integrin and/or PI3K.

## Methods

### Cell lines and culture conditions

NIH-3T3 fibroblasts were purchased from the American Type Culture Collection (ATCC), Manassas, VA and pre-conditioned (using 10% fetal bovine serum (FBS)) for 3D matrix production as published [47]. Primary tumor-associated fibroblasts were obtained as described [44], and used for a maximum of 8 passages. Breast epithelial MCF-10A and MDA-MB-231 cells were purchased from ATCC, while modified MCF-7 were a gift from Dr. V. Craig Jordan, FCCC Philadelphia, PA [49]. Fibroblasts were maintained in high glucose Dulbecco's modified Eagle's medium (DMEM; Mediatech Inc., Manassas, VA) containing 10% FBS (Hyclone, South Logan, UT), MCF-10As in high calcium DMEM with 5% horse serum (Invitrogen, Carlsbad, CA), while MCF-7 and MDA-MB-231 in RPMI-1640 (Mediatech, Inc., Manassas, VA) with 10% FBS. In addition, MCF-7 medium was complemented with 10 mM MEM Non-Essential Amino Acids Solution (NEAA, Mediatech, Inc., Manassas, VA), and with 10 ug/ml Bovine Pancreas Insulin from Sigma Aldrich (St. Louis, MO). All media were complemented with 100 U/ml penicillin, 100 μg/ml streptomycin, and 2 mM L-glutamine. Cells were cultured at 37°C in a humidified atmosphere of 5% CO_2_.

### Inhibitors

Functional blocking anti beta1- integrin antibody, mAb13 [50] used at a concentration of 50 μg/ml, was a gift from Dr. Kenneth Yamada (NIH/NIDCR, Bethesda, MD). Wortmannin, used at either 10 or 50 nM, and both negative controls DMSO and purified Rat IgG were purchased from Sigma Aldrich (St. Louis, MO).

### Production of fibroblast-derived 3D matrix

Murine embryonic fibroblasts, NIH-3T3s, were cultured in high glucose Dulbecco's modified Eagle's medium containing 10% fetal bovine serum for a minimum of 20 passages before matrix production to overcome their normal contact growth inhibition [47]. The resultant, pre-conditioned fibroblasts-derived 3D ECMs have been shown to be reminiscent of both *in vivo *and *in vitro *early stromal ECMs [44] and are characterized, among others, by random organization of fibronectin fibers. Therefore, matrices derived from these fibroblasts are referred to in the manuscript as "early" or "control" matrices. On the other hand, "tumor-associated matrices" were obtained from one, out of four different fibroblastic cell lines, isolated from advanced ("late") two-staged chemical carcinogenic-induced murine squamous cell carcinomas [44].

As previously published, the resultant "late" matrices are reminiscent of *in vivo *desmoplastic ECMs which are characterized, among others, by a parallel patterned matrix fiber organization [44]. The above-mentioned "control" and "tumor-associated" fibroblasts were induced to secrete and organize their own *in vivo*-like 3D matrices as described [44,46,47]. Briefly, 250,000 cells/ml were plated on chemically cross-linked gelatin and supplemented every 48 h with 50 μg/ml L-ascorbic acid (Sigma-Aldrich, St. Louis, MO). After 5–7 days, 3D matrices were cleared from cellular components by treatment with 0.5% (v/v) Triton X-100 and 20 mM NH_4_OH (Sigma Aldrich, St. Louis, MO). Resultant 3D matrices were stored at 4°C in PBS containing 100 U/ml penicillin and 100 μg/ml streptomycin. Every batch of matrices was characterized for quality control; matrices needed to be thicker than 7 μm while both types of ECMs were required to display the fiber organization characteristics associated with *in vivo *ECMs (as mentioned above).

### Cell growth assay

Cell growth was evaluated using Alamar Blue (Invitrogen, Carlsbad, CA), according to the manufacturer's instructions. Briefly, cells were plated at a density of 5,000 or 10,000 cells/well in 48- or 24-well plates and incubated for 24, 48 or 72 h prior to 10% (v/v) Alamar Blue treatment. After 4 h, changes in fluorescence (λ = 535/595) were measured using a SpectraFluor Plus (Tecan, San Jose, CA) plate reader. Wells void of cells were used as negative controls. All experiments were performed a minimum of three times in triplicates.

### Western blot analysis

1 × 10^5 ^cells were pre-incubated with inhibitor(s) or controls (see **Inhibitors **above) in their respective media and rotated for 15 min at 37°C. Cells were cultured overnight in the assorted matrices and lysates were prepared as previously described [45,51]. Proteins were resolved on SDS-PAGE using Tris-glycine 8–16% gels (Invitrogen, Carlsbad, CA) and transferred to PVDF membranes (Millipore, Bedford, MA). Primary antibodies were: anti-GADPH (1:5000, Chemicon Int, Millipore, Bedford, MA), anti-E-cadherin (1:2000, Abcam Inc, Cambridge, MA), anti-vimentin (1:3000, Sigma Aldrich, St. Louis, MO), anti-Akt (1:2000, BD Bioscience, San Jose, CA), anti-pAktS^473 ^(1:1000, Cell Signaling, Beverly, MA), anti-FAK (1:2000, Upstate, Millipore, Bedford, MA), and anti-pFAKY^397 ^(1:1000, Biosource, Camarillo, CA). Secondary antibodies were: goat anti-rabbit and anti-mouse conjugated to IRDye800 and IRDye680 (1:15,000 LI-COR, Lincoln, NE) for infrared scanning.

### Cell invasion within fibroblast-derived 3D matrices

Assays were conducted as previously described [48], with minor modifications. Briefly, 12 well plates containing assorted 3D matrices were used and 8,000 MDA-MB-231 cells were cultured overnight in the presence or the absence of inhibitors or respective negative controls (for details see **Inhibitors **above). Fresh medium containing 25 mM HEPES buffer was added, and cells were allowed to incubate for 1 h at 37°C. Using a motorized XYZ stage (Optical Apparatus Co., Ardmore, PA), five random acquisition points were pre-determined for each experimental well. Low throughput movies were created where each pre-set location was photographed every 10 min for a period of 6 h using an environmentally controlled Nikon TE-2000U wide field inverted microscope (Optical Apparatus Co., Ardmore, PA) with a Roper Scientific Cool Snap HQ camera rendering 5 time-lapse movies per well for a total of 60 movies per experimental plate. Non-dividing and non-clustered cells that remained within the field of view for the entire 6 h period were tracked using the "tracking objects" function in MetaMorph offline 7.0r4. The software rendered a set of 37 X,Y coordinates per recorded cell representing the location of the cell at every 10 minute time segment. These data sets were transferred to Microsoft Excel spreadsheets that were designed to automatically determine velocities (microns per hour), directionality, relative track orientation and trajectory length for each cell (see details below). All experiments were performed a minimum of two times in duplicates rendering no less than 10 movies per condition tested.

### Directionality assay

To evaluate the directionality of cells, trajectory angles of each cell with respect to the X axis were determined at 10 minute intervals. Angle values were obtained using the "Tracking objects" function of the MetaMorph software. This measurement rendered an average of 36 directional angles per cell, from which the mode angle (most common angle) was identified. Next, individual angles were rounded to the nearest 5^th ^degree, and compared to the identified mode angle. The experimental output per cell was determined as the percentage of angles that were within 5 degrees from the identified mode.

### Relative track orientation assay

The angle between the cell-track and the X-axis was determined based on X,Y coordinates obtained from MetaMorph software, resulting in a single angle per cell-trajectory. The resultant cell-track angles for each movie were rounded to the nearest 20^th ^degree in order to identify a mode (most common) angle per movie. In order to normalize the data, all modes were arbitrarily set as 0°, and the original tracks (per-movie) were rotated accordingly. The rotated track angles were rounded (de novo), to the 10^th ^degree. Movies in which no mode angle could be calculated were not rotated. In addition, only movies containing more than three cells were included in the study. Numbers indicating the percentages of cell-track angles within 20° from the mode (most common) angle per experimental condition, as well as total counts, were used to create the corresponding figures and tables presented.

### Cell morphology analysis

The assay was conducted as described [46], with minor modifications. In short, the first image from each time-lapse series acquired above (see Cell invasion within fibroblast-derived 3D matrices) was used for this assay. Cell contours were manually traced by a blinded individual and recognized by MetaMorph 7.0r4 software using "automatic threshold light objects" function. The resultant objects were then subjected to "integrated morphometry analysis" to obtain "elliptical form factor" (EFF) measurements. EFF is defined by the ratio of the "length" (long axis) over "breath" (short axis) of the cell; round objects present EFF = 1 while EFF > 1 represent spindled morphologies. All experiments were performed a minimum of two times, in duplicates rendering no less than 10 images (1 per movie) per condition tested.

### Statistical analyses

All statistical analyses were performed using the Mann-Whitney test and calculated by the Instat Statistical Software (GraphPad Software, San Diego, CA).

## Results

### Staged fibroblast-derived 3D ECMs do not impart a preferential growth environment to normal or tumorigenic breast epithelial cells

**Figure 1:**
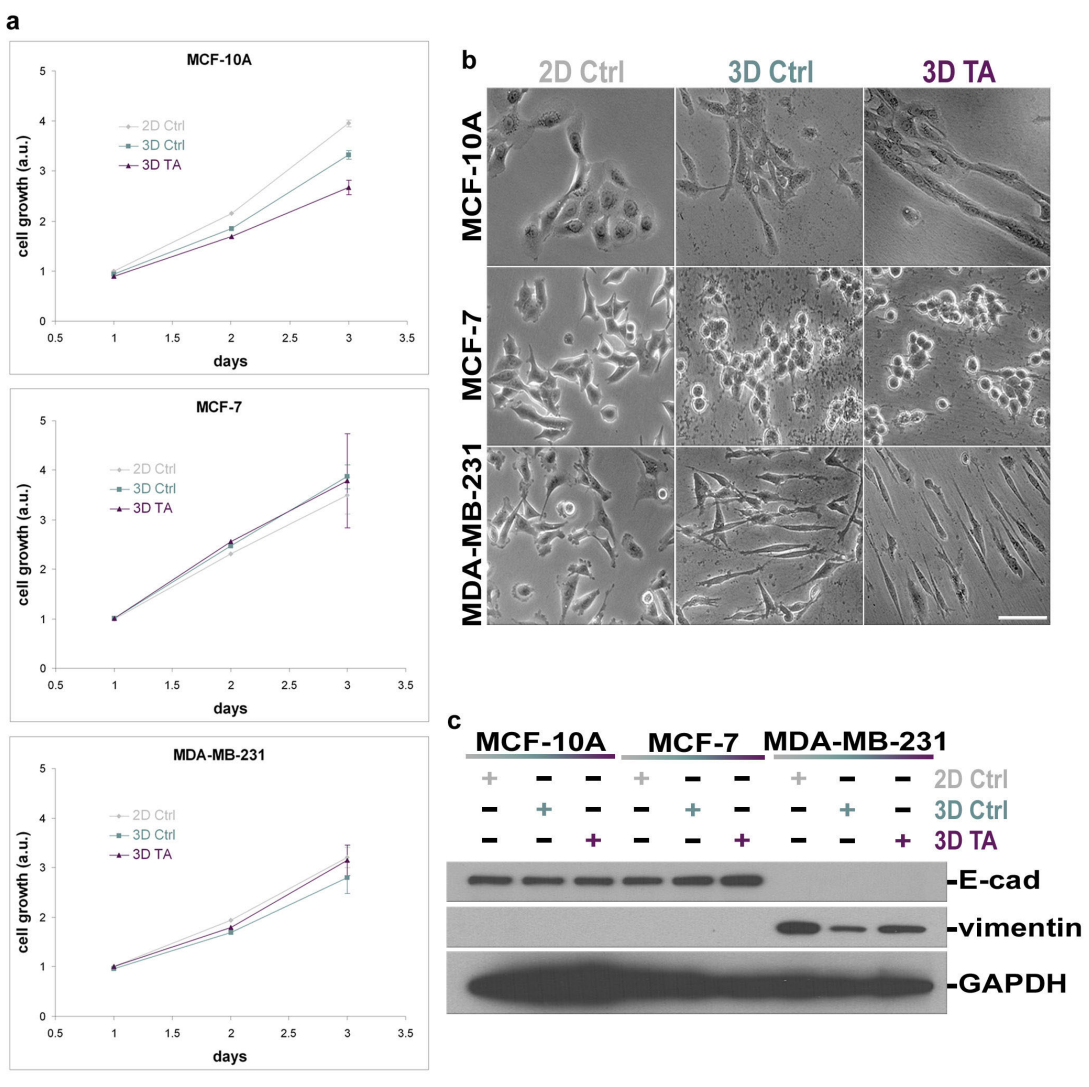
**MCF-10A, MCF-7 and MDA-MB-231 exhibit different cell behaviors in response to culturing on 2D or in staged 3D ECMs**. The stated cells were cultured on 2D (**2D Ctrl**), within 3D control (**3D Ctrl**), or within tumor-associated 3D ECMs (**3D TA**). **a**. Growth rates were calculated every 24 h for a period of 72 h. Note that 3D ECMs appear to be growth inhibitory only in MCF-10A cells with slower growth rates induced by tumor-associated 3D matrices. **b**. phase contrast transmitted light micrographs depicting changes in cell morphologies of the various cell lines in response to the 2D and 3D substrates are shown; scale bar represents 75 μm. **c**. Western blot of lysates obtained from the various cell lines cultured in the assorted substrates showing expressions of epithelial marker E-cadherin (**E-cad**), mesenchymal marker **vimentin **and loading control glyceraldehyde 3-phosphate dehydrogenase (**GAPDH**). Note that the 3D ECMs did not alter the original epithelial or mesenchymal characteristics of the assorted cells.

To test whether our stromal staged mesenchymal ECMs induce breast epithelial cell growth, we cultured MCF-10A, MCF-7 or MDA-MB-231 cells in 2D conditions, control (resembling early) or tumor-associated (resembling late) 3D ECMs and quantitatively measured their growth rates during a period of 3 days. Our measurements showed that MCF-10A had a small, yet highly significant, preference to grow on 2D conditions compared to control 3D ECMs (1.1 fold, with P < 0.0001) or tumor-associated 3D ECMs (1.3 fold, P < 0.0001). The differences in growth rates on the 3D matrices were also highly significant (P < 0.0001). In contrast, tumorigenic MCF-7 and invasive MDA-MB-231 cells showed no significant differences in their growth rates under all conditions tested (Figure 1a). These results suggested that fibroblast-derived 3D ECMs differentially regulate the growth rates of some, but not all, epithelial cells.

### Effects of staged 3D ECMs in breast epithelial cell morphologies

Since different cell morphologies have been associated with many tumorigenic characteristics [52,53] and with a variety of invasive strategies [26,27,54], we proceeded to ask whether staged 3D matrices could differentially influence breast epithelial cellular morphologies. MCF-10A, MCF-7 or MDA-MD-231 cells were cultured overnight on 2D, within 3D control, or within tumor-associated 3D ECMs, and their morphologies were perceptibly assessed using transmitted light microscopy. While cell morphologies were similar on 2D cultures, Figure 1b shows that all cells presented altered morphologies when comparing 2D vs. 3D substrates. Interestingly, while both MCF-10A and MCF-7 seemed to aggregate in cell clusters within 3D microenvironments, their morphologies were very different; MCF-10A became spindle-like, while MCF-7 presented relatively rounded and less spread morphology. In comparison, tumorigenic and invasive MDA-MB-231 cells, adopted spindled morphology in both staged 3D ECMs, as expected from these invasive mesenchymal-like cells. Moreover, matrix-induced morphologies of these epithelial-to-mesenchymal transitioned cells appeared to be enhanced (more spindled) within tumor-associated 3D ECMs when compared to 3D control (see measurements below). It is important to note that in addition to the spindled morphology, MDA-MB-231 cells remained unclustered as opposed to the other two cell lines tested.

The epithelial or mesenchymal nature of MCF-10A and MCF-7 or MDA-MB-231 [55] and the fact that 3D matrices do not affect their epithelial vs. mesenchymal characteristics were confirmed by Western blot analyses using epithelial marker E-cadherin and mesenchymal marker vimentin (Figure 1c).

### Staged 3D matrices effectively support single-cell MDA-MB-231 and clustered-cell MCF-10A invasion

Real time motility (on control 2D), as well as invasive (within staged fibroblast-derived 3D matrices) 6 h period assays, were used to observe correlations between invasive behaviors and substrate-induced morphologies of the cells used in this study. MCF-10As were observed to be motile, on 2D control, and invasive, in both staged 3D ECMs. In addition, although all cells were seeded as individual cells, MCF-10As seemed to cluster and invade through the staged 3D matrices as aggregates or groups consisting of several cells (Additional file 1; Movie 1). In comparison to MCF-10A, MCF-7 and MDA-MB-231 were not very motile under 2D conditions (see Additional files 2 and 3; Movies 2 and 3). However, while both MCF-10A and MCF-7 cells clustered in 3D matrices, MCF-7 did not present any invasive characteristics (Additional file 2; Movie 2). Alternatively, epithelial to mesenchymal transitioned MDA-MB-231 cells presented apparent mesenchymal-like invasive behaviors (e.g., relatively directional cell movement through 3D matrices, see Additional file 3; Movie 3 plus measurements below). These results imply that matrix-induced cell morphologies could be suggestive of breast cancer cell invasive occurrence (compare Figure 1b with Additional files 1, 2 and 3; Movies 1–3).

### Tumor-associated 3D matrix supports sustained Akt/PKB activity regulated by both PI3K and beta1-integrin

**Figure 2:**
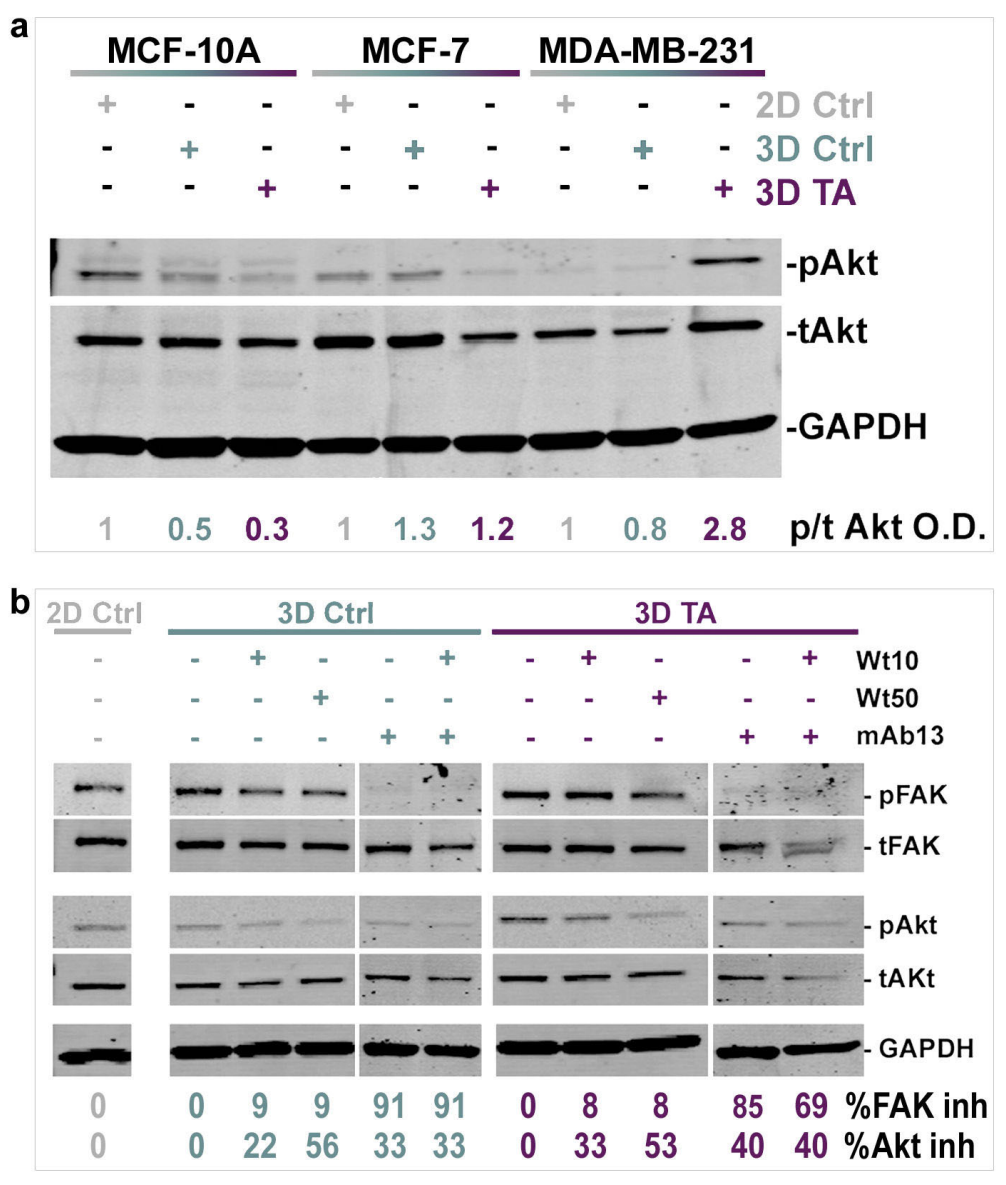
**Tumor-associated 3D matrix induced Akt/PKB activity in MDA-MB-231 is regulated by both PI3K and beta-1 integrin**. **a**. Representative Western blot showing levels of pAktS^473 ^(**pAkt**), total Akt (**tAkt**), and Glyceraldehyde 3-phosphate dehydrogenase (**GAPDH**), used as endogenous protein loading control, in lysates from MCF10A, MCF7 and MDA-MB231 cultured on 2D or within staged 3D ECMs. Specific Akt/PKB activity was calculated as the ratio of the scanned optical density (O.D.) of pAktS^473^/totalAkt (**p/tAkt O.D.**). The calculated activity ratios were normalized to ratios obtained for each cell line cultured on 2D control conditions and were individually assigned as one arbitrary unit. Note that tumor-associated 3D matrix induces high specific Akt/PKB activity levels in MDA-MB-231 but not in MCF-10A or MCF-7 cells. **b**. Lysates from MDA-MB-231 cells cultured on 2D or in staged 3D ECMs in the presence and/or absence of 10 and 50 nM Wortmannin (**Wt10 **and **Wt50**) and/or 50 μg/ml mAb13 (**mAb13**) were proved by Western immunoblotting using antibodies against pFAKY^397 ^(**pFAK**), total FAK (**tFAK**), pAKTS^473 ^(**pAkt**), total Akt (**tAKT**) and Glyceraldehyde 3-phosphate dehydrogenase (**GAPDH**). The percentages of inhibition of FAK (**%inh FAK**) and Akt/PKB (**%inh Akt**) activities are shown and were calculated using the corresponding O.D. ratios: pFAKY^397^/totalFAK and pAktS^473^/totalAkt. Note that while Wortmannin inhibited Akt/PKB activity, mAb13, alone or in combination with Wortmannin, inhibited both FAK and Akt/PKB activities.

Since the serine/threonine protein kinase Akt/PKB, has been associated with both matrix induced cell survival and invasion [56,57], matrix-induced Akt/PKB activity levels were tested. For this, MCF-10A, MCF-7 or MDA-MD-231 were cultured on 2D conditions, or within staged 3D ECMs for a period of 18h. Cells were lysed and levels of active (pAktS^473^) and total Akt/PKB (tAkt) protein populations were assessed using Western blot analyses (Figure 2a). In control, compared to tumor-associated 3D ECMs, constitutive Akt/PKB (pAkt S^473^/tAkt) activity levels in MCF-10A and MCF-7 were either down regulated or remained unchanged (O.D = 0.5 and. 0.3 vs. 1.3 and 1.2, respectively), while these levels were clearly up-regulated in MDA-MB-231 cells (O.D = 0.8 and 2.8). These results suggested that tumor-associated 3D matrices, but not 3D controls, constitutively activated Akt/PKB in MDA-MB-231 but not in MCF-10A or MCF-7 cells.

Next, tumor-associated 3D matrix induced pathways responsible for the observed constitutive activity of Akt/PKB in MDA-MB-231 cells were analyzed. Since Phosphoinositide-3 kinase (PI3K) and beta1-integrin pathways have been associated with increased levels of Akt/PKB activities [56,58,59], these two Akt/PKB regulators were selectively and/or collectively inhibited while levels of Akt/PKB activity, and of beta1-integrin effector, focal adhesion kinase (FAK), were assessed. Untreated 2D conditions were used for normalization purposes and assigned a value of one arbitrary unit. Results show that PI3K inhibitor, Wortmannin, effectively inhibited Akt/PKB activity, while anti beta1-integrin functional blocking antibody, mAb13 [50], inhibited both Akt/PKB and FAK activities induced by the two staged 3D ECMs. A representative Western blot with indicated percentages of FAK and Akt/PKB inhibitions is displayed in Figure 2b. In addition, Figure 2b shows that both FAK and Akt/PKB activities were also effectively inhibited when combination treatments of Wortmannin and mAb13 were used. Consequently, both Wortmannin and mAb13 were used to analyze a variety of staged matrix-induced cell responses such as cell morphology and various aspects of cell invasion (e.g., velocity, directionality and relative track orientation) in the second part of this study.

### PI3K and beta1-integrin differentially regulate staged 3D matrix-induced MDA-MB-231 spindled morphologies

**Figure 3:**
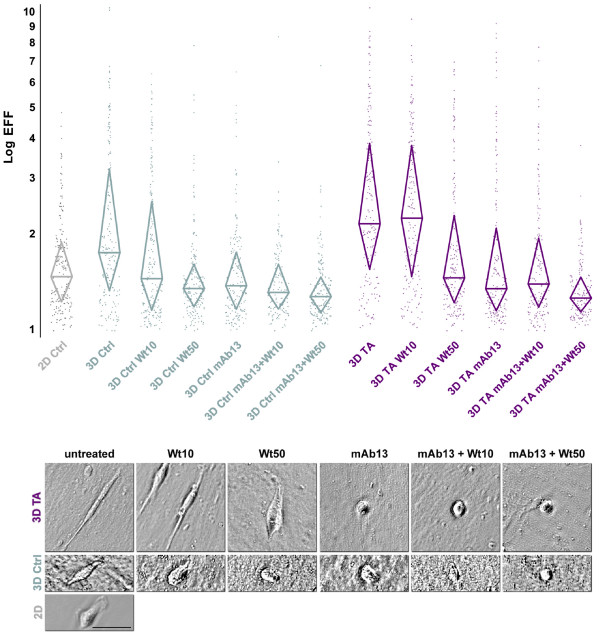
**PI3K and beta1-integrin differently regulate staged 3D ECM induced MDA-MB-231 morphologies**. MDA-MB-231 cells were cultured overnight on 2D or within staged 3D ECMs, control and tumor-associated matrices, in the presence and/or absence of 10 and 50 nM Wortmannin and/or 50 μg/ml mAb13. **a**. Individually scattered dots represent elliptical form factors (**EFF**). Upper and lower borders of diamond shapes show 75 and 25 percentile EFF populations, respectively while diamond widths marked with a horizontal line denote median EFF values. **b**. Representative transmitted light micrographs depicting one cell for each stated experimental condition. Scale bar indicates 50 μm. Note that median EFF increases in response to tumor-associated 3D ECMs, and that these EFFs can be inhibited by high Wortmannin or mAb13 treatments while combinations of both inhibitors seemed to inhibit better than each of these alone. See Table 1 for detailed quantitative data, Table 2 for statistical information and Additional Files 4, 5 and 6 (Movies 4–6) for additional examples.

To better understand tumor-associated induced invasive breast cancer cell responses, the morphological features of MDA-MB-231 cells in control (early) vs. tumor-associated (late) 3D ECM under PI3K, and/or beta1-integrin inhibition were quantified. For this, MDA-MB-231 cells were plated overnight within staged 3D ECMs in the presence or absence of Wortmannin and/or mAb13 (DMSO and/or rat antibody were used as negative controls; see Methods for details). As expected, results shown in Figure 3 and quantified in Table 1 confirmed that increased spindled morphologies, measured as median elliptical form factor (mEFF), were indeed observed in tumor-associated when compared to control 3D ECMs (mEFF = 2.2 and 1.7, respectively) while Table 2 showed that this increase was statistically significant (P < 0.0001). Moreover, in control 3D matrices, 10 and 50 nM concentrations of PI3K inhibitor Wortmannin show mEFF ratios of 1.5 vs. 1.4, which were statistically lower than mEFF ratios in untreated 3D control (P = 0.0016 and < 0.0001, respectively), yet not significantly different from each other (P = 0.1902). In contrast, in tumor-associated 3D ECMs, low concentrations of Wortmannin appeared to have no effect (mEFF = 2.3 with P = 0.5975) while higher concentrations were needed to attain significant inhibitory mEFF ratios (mEFF = 1.6 with P < 0.0001) when compared to untreated control. Interestingly, blocking of beta1-integrin function by treating cells with mAb13, showed a more effective inhibition of mEFF ratios in tumor-associated (36% inhibition) than in control 3D ECMs (18% inhibition), reaching 1.4 mEFF ratios with statistical P values smaller than 0.0001 in both cases. On the other hand, 50 nM Wortmannin and mAb13 showed no significant differences (mEFF = 1.4 with P = 0.2022) when compared to each other in control 3D ECMs. The effects of these treatments were modest (mEFF = 1.6 and 1.4, respectively) yet still significantly different from each other (P = 0.0005) in tumor-associated 3D ECMs. Since the most effective mEFF inhibitory effects were attained when both PI3K and beta1-integrin were blocked simultaneously, results suggested that both pathways played a role in regulating 3D matrix induced cell morphologies. However, results also suggested that while beta1-integrin more effectively regulated tumor-associated 3D matrix induced MDA-MB-231 spindled morphologies, control 3D matrix-induced morphology seemed to be more sensitive to PI3K inhibition.

**Table 1:** 3D matrix induced morphology measurements; EFF ±

substrate	2D	control 3D						tumor-associated 3D					
treatment	Ctrl	Ctrl	Wt 10 nM	Wt 50 nM	mAb13	mAb13 +Wt 10 nM	mAb13 +Wt 50 nM	Ctrl	Wt 10 nM	Wt 50 nM	mAb13	mAb13 +Wt 10 nM	mAb13 +Wt 50 nM
Median (mEFF)	1.5	1.7	1.5	1.4	1.4	1.3	1.3	**2.2**	**2.3**	**1.6**	**1.4**	**1.4**	**1.3**

Sample Size	172	251	192	294	173	130	221	**292**	**210**	**290**	**206**	**181**	**248**

Std. Err	0.1	0.1	0.1	0.1	0.1	0.1	0.0	**0.1**	**0.1**	**0.1**	**0.1**	**0.1**	**0.0**

^±^EFF = elliptical form factor ratio.

**Table 2:** morphology P values

Sample	2D	3D	Wt 10 nM	Wt 50 nM	mAb13	mAb13 +Wt 10 nM	mAb13 Wt 50 nM
untreated							
Ctrl	< 0.0001***	-	0.0016**	< 0.0001***	< 0.0001***	< 0.0001***	< 0.0001***
TA	< 0.0001***	< 0.0001***	**0.5975**	< 0.0001***	< 0.0001***	< 0.0001***	< 0.0001***

Wt 10 nM							
Ctrl	-	-	-	**0.1902**	0.0221**	0.0009***	< 0.0001***
TA	-	-	-	< 0.0001***	< 0.0001***	< 0.0001***	< 0.0001***

Wt 50 nM							
Ctrl	-	-	-	-	**0.2022**	0.0042**	< 0.0001***
TA	-	-	-	-	0.0005***	< 0.0001***	< 0.0001***

mAb13							
Ctrl	-	-	-	-	-	0.1687	0.0005***
TA	-	-	-	-	-	0.4596	0.0108*

mAb13 + Wt 10 nM							
Ctrl	-	-	-	-	-	-	0.0686
TA	-	-	-	-	-	-	0.0742

P values were obtained using Mann-Whitney test. Non-significant values are underlined while relative significance levels are designated as extremely***, very**, or significant*. Bolded data represent conditions in which differences were observed between control and tumor-associated 3D matrix induced EFF due to stated treatments.

### PI3K and beta1-integrin differentially regulate staged 3D matrix induced velocity, directionality and relative track orientation in MDA-MB-231 cells

Next, we decided to analyze the staged 3D matrix induced invasive characteristics of MDA-MB-231 cells while questioning the roles that PI3K and beta1-integrin play in these invasive behaviors. Three different aspects of staged 3D matrix induced single cell invasion, velocity, directionality and relative track orientation, were measured in response to the presence or absence of PI3K and beta1-integrin inhibitors (Wortmannin and/or mAb13, respectively). For this, six-hour time-lapse videos were created in a semi-high throughput manner thus simultaneously recording the cell's motile behaviors in response to the various experimental settings (see Methods). Additional files 4, 5 and 6 (Movies 4, 5 and 6) contain a representative example for each condition tested.

#### Velocity

**Figure 4:**
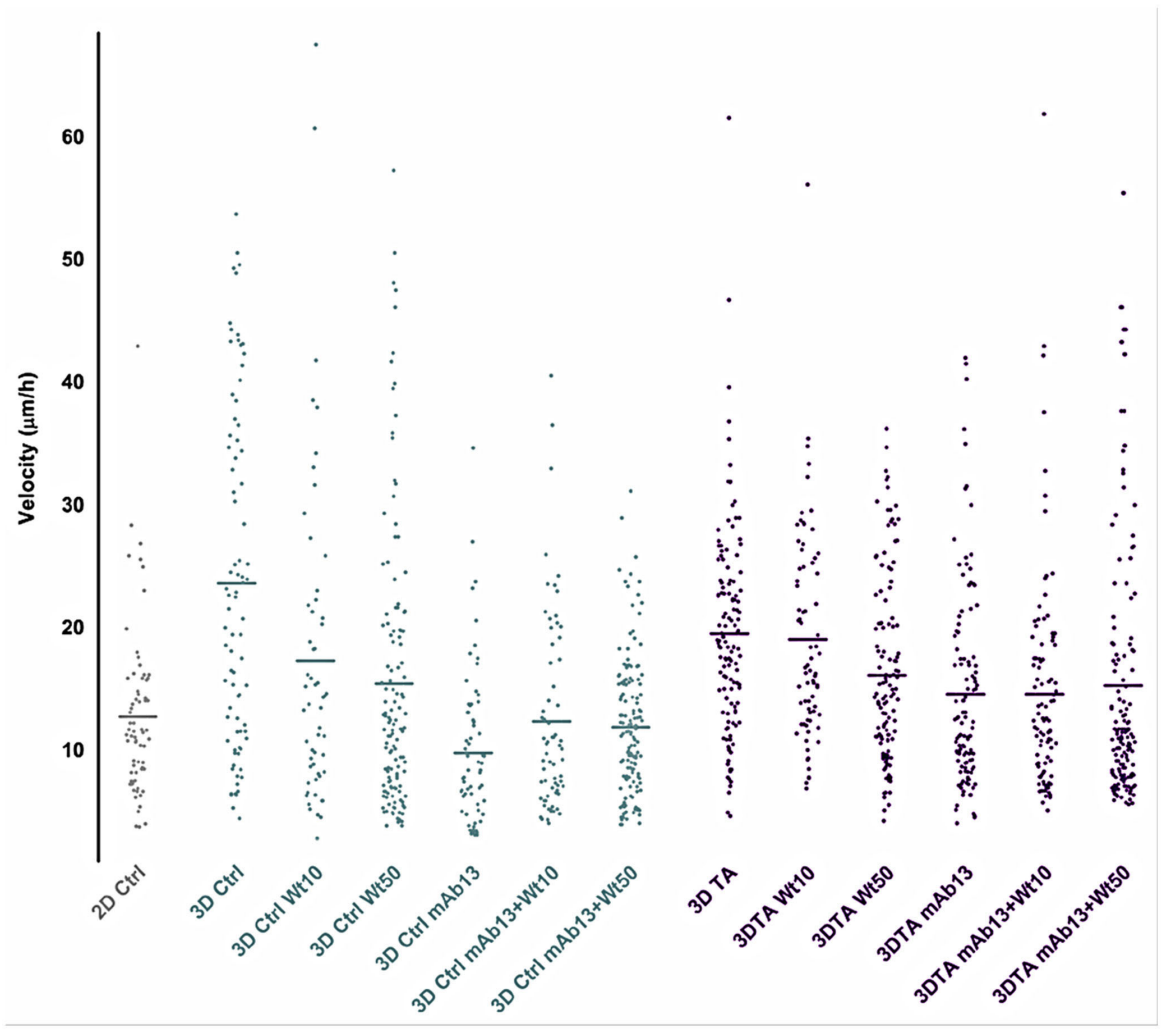
**Staged 3D ECMs induce relatively fast MDA-MB-231 invasion that is differentially regulated by PI3K and beta1-integrinpathways**. Time-lapse assays spanning 6 h were carried out as described in Methods, to determine the velocity of MDA-MB-231 cells cultured on 2D or within staged 3D ECMS in the presence and/or absence of 10 and 50 nM Wortmannin and/or 50 μg/ml mAb13. Cell velocities, calculated in microns per hour (μm/h), were plotted as individual dots while mean velocities are marked with horizontal lines (see Tables 3 and 4 for quantitative details). Note that Wortmannin and mAb13 treatments induced different effects in velocities of cells invading through control vs. tumor-associated 3D ECMs.

Cell velocities were calculated as microns per hour. As shown in Figure 4 and summarized in Tables 3 and 4, the velocity of cell displacement was significantly slower in 2D when compared to staged control and tumor-associated 3D ECMs (12 μm/h vs. 23 μm/h and 19 μm/h, respectively with Ps < 0.0001). In addition, the relatively high velocities observed in both control and tumor-associated 3D ECMs were not significantly different from each other (P = 0.1606). Measurements under PI3K blockage showed that while 10 nM Wortmannin effectively inhibited cell velocity in 3D control, this concentration had no effect in tumor-associated 3D ECMs (17 μm/h and 19 μm/h with P = 0.0023 and 0.5286, respectively). Higher Wortmannin concentrations (50 nM) slightly, yet not significantly, further inhibited cell velocities induced by 3D control, while this concentration caused a statistically significant inhibition of velocity induced by tumor-associated 3D ECMs (15 vs. 16 μm/h with P = 0.2859 vs. 0.0114, respectively). Interestingly, mAb13 treatments, blocking the function of beta1-integrins, were found to have greater effects in control than in tumor-associated 3D ECMs (9 μm/h vs. 14 μm/h representing 60% vs. 26% inhibition compared to untreated, respectively). When both drugs were used in combination, cells in control 3D ECMs were significantly faster compared to mAb13 alone (12 μm/h and 11 μm/h with P = 0.0348 and 0.0015, respectively). In comparison, in tumor-associated matrices no additional effects were observed (14 μm/h and 13 μm/h with P = 0.8733 and 0.1978, respectively). The results suggested that beta1-integrin regulates the velocity of MDA-MB-231 cells, and that PI3K inhibition somewhat abolished the beta1-integrin regulatory effect in control 3D ECMs, while these two pathways seemed to work in tandem in the regulation of cell velocity induced by tumor-associated 3D ECMs.

**Table 3:** matrix induced velocity (μm/h)

substrate	2D	control 3D						tumor-associated 3D					
treatment	Ctrl	Ctrl	Wt 10 nM	Wt 50 nM	mAb13	mAb13 +Wt 10 nM	mAb13 +Wt 50 nM	Ctrl	Wt 10 nM	Wt 50 nM	mAb13	mAb13 +Wt 10 nM	mAb13 +Wt 50 nM
Mean	12	23	17	15	9	12	11	**19**	**19**	**16**	**14**	**14**	**13**

Sample Size	69	83	56	130	68	69	148	**130**	**74**	**129**	**115**	**99**	**150**

Std. Err	1	2	2	1	1	1	0	**1**	**1**	**1**	**1**	**1**	**1**

**Table 4:** velocity P values

Sample	2D	3D	Wt 10 nM	Wt 50 nM	mAb13	mAb13 +Wt 10 nM	mAb13 Wt 50 nM
untreated							
Ctrl	< 0.0001***	-	0.0023**	< 0.0001***	< 0.0001***	< 0.0001***	< 0.0001***
TA	< 0.0001***	0.1606	**0.5286**	0.0003***	< 0.0001***	< 0.0001***	< 0.0001***

Wt 10 nM							
Ctrl	-	-	-	**0.2859**	< 0.0001***	0.0190*	0.0193*
TA	-	-	-	0.0114*	< 0.0001***	< 0.0001***	< 0.0001***

Wt 50 nM							
Ctrl	-	-	-	-	0.0002***	**0.1075**	**0.1571**
TA	-	-	-	-	0.0304*	0.0247*	0.0002***

mAb13							
Ctrl	-	-	-	-	-	0.0348*	0.0015**
TA	-	-	-	-	-	**0.8733**	**0.1978**

mAb13 + Wt 10 nM							
Ctrl	-	-	-	-	-	-	0.4986
TA	-	-	-	-	-	-	0.2165

P values were obtained using Mann-Whitney test. Non-significant values are underlined while relative significance levels are designated as extremely***, very**, or significant*. Bolded data represent conditions in which differences were observed between control and tumor-associated 3D matrix induced velocity due to stated treatments.

#### Directionality

**Figure 5:**
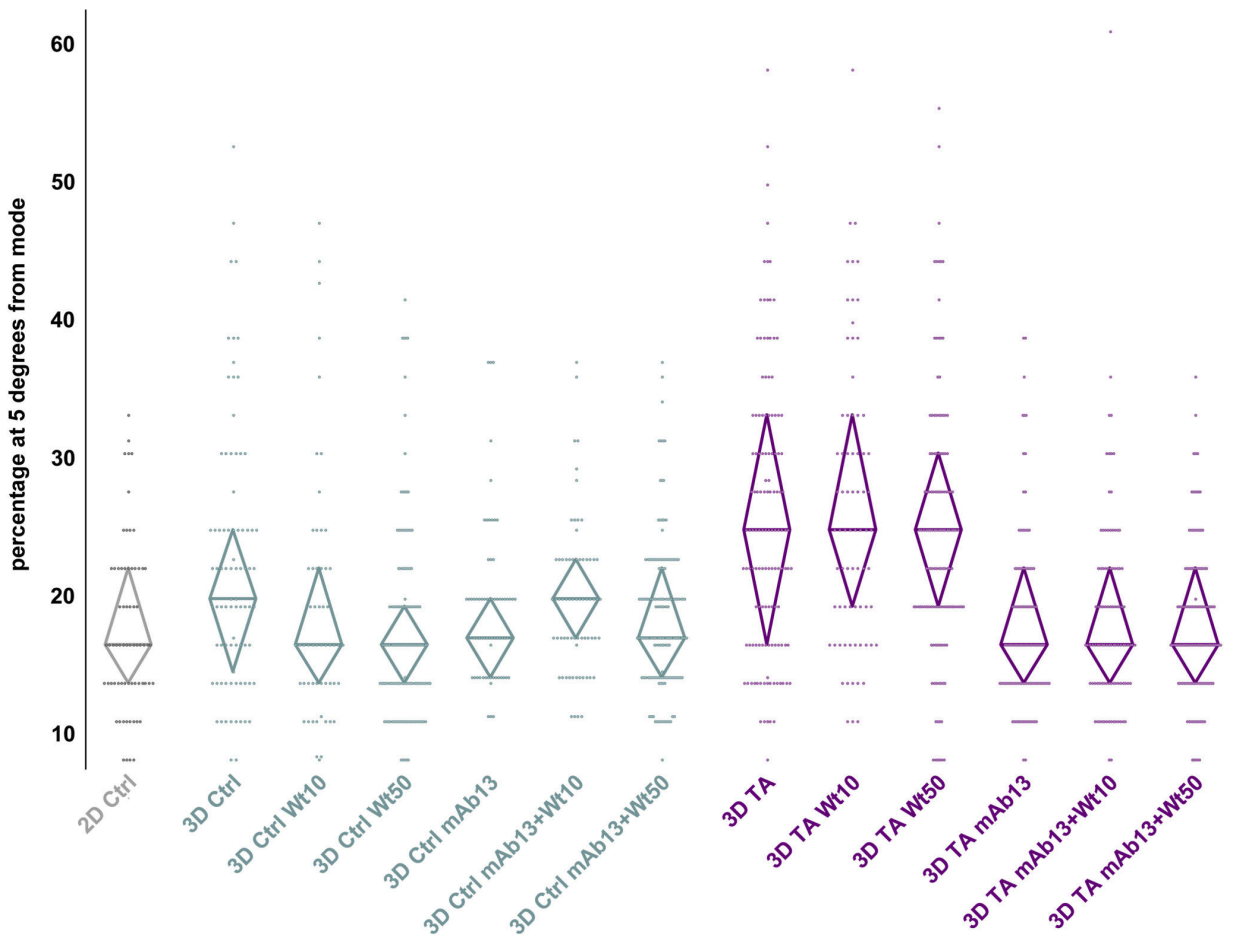
**Tumor-associated 3D ECMs induce directional MDA-MB-231 invasion regulated by beta1-integrin but not PI3K**. Time-lapse assays were carried out to determine the directionality of MDA-MB-231 cells in the presence and/or absence of 10 and 50 nM Wortmannin and/or 50 μg/ml mAb13. Dots plotted in graph indicate percentages of angles positioned within 5° from the identified mode angle per cell (data was rounded and therefore appears organized (for additional details, see Material and Methods)). Upper and lower borders of diamond shapes show 75 and 25 percentile populations, respectively, while diamond widths marked with a horizontal line, mark median percentages at 5° variance from the mode angle direction. Note that when compared with 2D and 3D matrices, tumor-associated 3D matrices induced greater degree of directionality, which was regulated by beta1-integrin but not by PI3K activity. See Tables 5 and 6 for statistical information.

Since we have previously shown that fibroblasts invade in a directional manner within control 3D ECMs as opposed to migrating randomly on 2D substrates [43], and in addition, directionality has been observed *in vivo *in highly metastatic mammary cells invading through mesenchymal stroma [25], directionality of cells was measured in staged, control vs. tumor-associated, 3D ECMs. Cell directionality, was assessed by measuring the angle of direction of each cell in segments spanning 10 minutes each. The percentages of cell-directions including angles within 5° from the mode angle, in each cell trajectory, were calculated and are shown in Figure 5 while a summary of the data is presented in Tables 5 and 6. Cells with higher percentage of similar angles represent persistent directional behaviors, while lower percentages indicate tracks that followed random directions. Results indicated that while cells moved randomly on 2D conditions (18%) they seemed to invade with a high degree of directionality through both control and tumor-associated 3D ECMs (22% and 26%). Moreover, this increase in directionality was significant between staged 3D matrices (P = 0.0109). Measurements performed under inhibitory PI3K conditions showed that while directionality was modestly to significantly decreased by 10 and 50 nM Wortmannin in control 3D matrices (19% and 18% with P = 0.0219 and < 0.0001, respectively), the inhibitor seemed to have no effect on directionality induced by tumor-associated 3D ECMs (26% and 25% with P = 0.6641 and 0.5551, respectively). On the other hand, while mAb13 seemed to have no significant effect on cellular directionality in the control (19% with P = 0.2395), it appeared to have a profound inhibitory effect in tumor-associated 3D ECMs (18% with P < 0.0001). Combinations of Wortmannin and mAb13 did not change observations obtained using mAb13 as a single treatment. Results suggested that beta1-integrin regulates tumor-associated 3D ECM-induced directional invasion of MDA-MB-231 cells, while PI3K somewhat regulated the directionality of these cells induced by control 3D ECMs.

**Table 5:** 3D matrix induced directionality (±%)

substrate	2D	control 3D						tumor-associated 3D					
treatment	Ctrl	Ctrl	Wt 10 nM	Wt 50 nM	mAb13	mAb13 +Wt 10 nM	mAb13 +Wt 50 nM	Ctrl	Wt 10 nM	Wt 50 nM	mAb13	mAb13 +Wt 10 nM	mAb13 +Wt 50 nM
Mean	18	22	19	18	19	20	19	**26**	**26**	**25**	**18**	**19**	**18**

Sample Size	69	83	56	130	68	69	148	**130**	**74**	**129**	**115**	**99**	**150**

Std. Err	1	1	1	1	1	1	0	**1**	**1**	**1**	**1**	**1**	**0**

^±^% of cells at 5° distance from mode angle

**Table 6:** directionality P values

Sample	2D	3D Ctrl	Wt 10 nM	Wt 50 nM	mAb13	mAb13 + Wt 10 nM	mAb13 Wt 50 nM
untreated							
Ctrl	< 0.0014**	-	0.0219*	< 0.0001***	**0.2395**	**0.6252**	0.0377*
**TA**	< 0.0001***	0.0109*	**0.6641**	**0.5551**	< 0.0001***	< 0.0001***	< 0.0001***

Wt 10 nM							
Ctrl	-	-	-	0.3691	**0.0545**	0.0109*	**0.1144**
**TA**	-	-	-	0.3983	< 0.0001***	< 0.0001***	< 0.0001***

Wt 50 nM							
Ctrl	-	-	-	-	0.0002***	< 0.0001***	0.0004***
**TA**	-	-	-	-	< 0.0001***	< 0.0001***	< 0.0001***

mAb13							
Ctrl	-	-	-	-	-	0.2808	0.5132
**TA**	-	-	-	-	-	0.4318	0.2500

mAb13 + Wt 10 nM							
Ctrl	-	-	-	-	-	-	0.0568
**TA**	-	-	-	-	-	-	0.8769

P values were obtained using Mann-Whitney test. Non-significant values are underlined while relative significance levels are designated as extremely***, very**, or significant*. Bolded data represent conditions in which differences were observed between control and tumor-associated 3D matrix induced directionality due to stated treatments.

#### Relative track orientation

**Figure 6:**
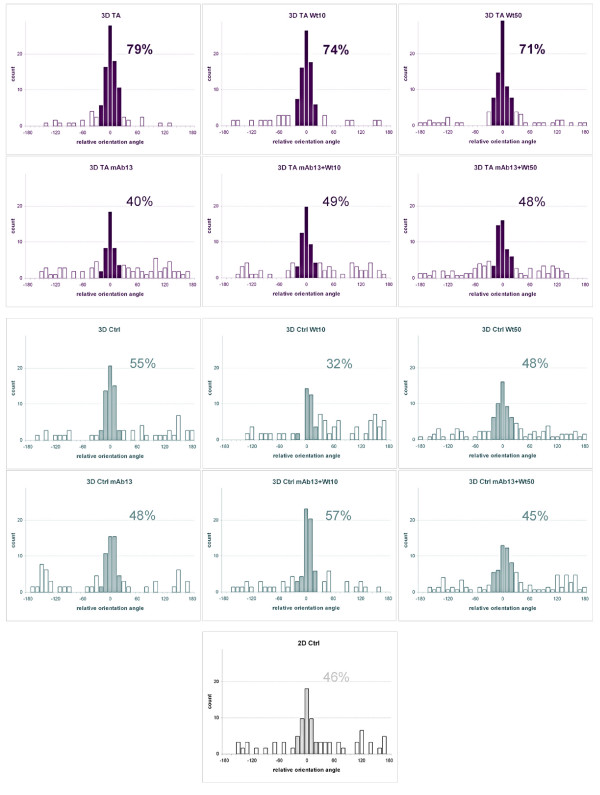
**Tumor-associated 3D ECMs induce relative organized MDA-MB-231 track orientations regulated by beta1-integrin but not by PI3K pathways**. Time-lapse assays were carried out using MDA-MB-231 cells cultured on 2D or within staged 3D ECMs in the presence and/or absence of 10 and 50 nM Wortmannin and/or 50 μg/ml mAb13. Representative trajectory tracks were attained using Microsoft Excel as described in Methods. Orientation angles of the trajectories relative to the X-axis were rounded to the nearest 20^th ^degree in order to identify a mode orientation angle. This mode angle was arbitrarily set as 0° and the original angles were rotated accordingly. Next, a new rounding of the rotated but otherwise intact data was performed only this time it was approximated to the 10^th ^degree (see Methods for additional details). The percentages of angles positioned within 20° variance from the mode angle are indicated in the figure. Note that tumor-associated 3D matrices support oriented cell invasive trajectories (greater than 70% organization) in a beta1-integrin but not PI3K dependent manner.

We have previously shown that similar to their *in vivo *counterparts, tumor-associated ECMs present parallel fiber patterns of organization [44]. In addition, it has been suggested that cells utilize matrix-patterned fibers for their mesenchymal type of invasive behavior [15,25,26]. Therefore, we proceeded to measure the relative track orientations of cells in all conditions. For this, the mode angle of cell-track orientation was calculated on each recorded region, and all cell tracks in this region were reoriented to fit a common arbitrary mode angle of 0°. Percentages of cell-tracks sharing common angles were plotted (Figure 6). Conditions inducing 70% or more counts at 20° distance from the mode angle were considered as "organized." The results show a high degree of orientation in cells invading through tumor-associated as opposed to control 3D matrices (79% vs. 55%, respectively). In addition, low or high levels of PI3K inhibition did not apparently affect the relative orientation of MDA-MB-231 cells invading through tumor-associated 3D ECMs (74% and 71%, respectively), while beta1-integrin inhibition effectively disorganized their relative orientation reaching only 40% of tracks at 20° variance from the mode. Moreover, the relative disorganized pattern seemed to prevail when mAb13 was used in combination with low or high concentrations of Wortmannin (49% or 48%, respectively). As expected, none of the treatments induced relative track orientation in control 3D ECM induced MDA-MB-231 invasion. The results presented herein suggested that tumor-associated, but not control 3D ECMs, induced an oriented parallel pattern of invasion in MDA-MB-231, and that this orientation is beta1-integrin dependent and PI3K independent.

## Discussion

It is well established that tumor-associated stromal ECMs influence tumorigenesis [60]. It has been shown that increase in collagen density, as seen in our late 3D matrices [44], supports tumor formation, invasion and metastases [61]. Moreover, it has been proposed that Activated Stromal Indexes, calculated as ratios between the degree of fibrous mesenchymal collagen and levels of a desmoplastic marker (alpha-smooth muscle actin), can be useful tools for assessing probable neoplastic prognostics [62]. In this study, we observed that assorted cells present different behaviors within reciprocal ECMs. Therefore, the results of this study suggest a need for cellular predisposition to invasion even though classic 2D culturing methods were found to be insufficient for predicting tumorigenicity of cells. This is particularly apparent when comparing 3D to 2D conditions. For example, in 2D conditions, the invasive potential of MCF-10A, MCF-7 and MDA-MB-231 cells could not be determined as they exhibited similar behaviors. Nevertheless, it was not surprising to discover that the stage of the 3D matrices further contributed to the induction of *in vivo-*like cell invasion.

**Figure 7:**
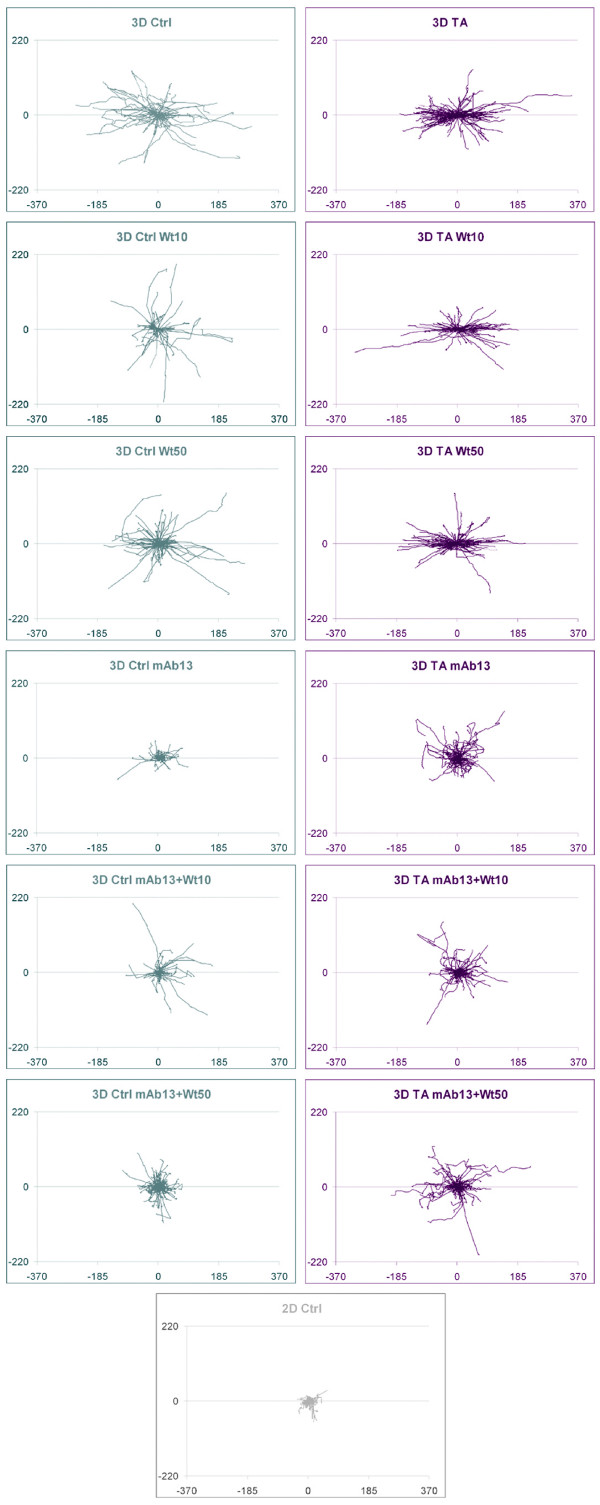
**PI3K and beta1-integrin pathways play different roles in regulating 3D matrix-induced breast cancer cell invasion**. Representative MDA-MB-231 trajectory tracks were attained as described in Methods. Orientation angles of the trajectories relative to the X-axis were rounded to the nearest 20^th ^degree in order to identify a mode orientation angle. This mode was arbitrarily set as 0° and the original angles were rotated accordingly (see Methods for additional details). The resulting tracks were plotted as if all shared a common origin to generate a star like pattern. Graph scales are shown in micrometers to appreciate distances travelled during the recorded 6 h periods. Note that cells invading through tumor-associated 3D ECMs appear to move relatively fast (length of tacks), directional (straightness of tracks) and with a relative greater level of common or parallel orientation (orientation of tracks near the X-axis) when compared to control 3D ECMs. In addition, inhibition of beta1-integrin function (mAb13) effectively blocked cell invasion through control 3D ECMs, as shown by the resulting relatively small star. In contrast, blockage of beta1-integrin activity in these invasive cells triggered a change in the invasive strategy induced by tumor-associated 3D ECMs. This observation is apparent by contemplating the relatively large stars with wiggle tracks, as opposed to straight tracks, that lack organization near the X-axis in all experimental data attained in the presence of mAb13.

It has previously been shown that matrix extracts obtained from murine mammary glands at various developmental stages, differently influence MDA-MB-231 invasion [63], and that both age and reproductive state of stroma, can differentially affect breast tumor development [64]. In addition, it has been established that diverse pathways affect distinct cell invasion strategies, such as "mesenchymal invasion," characterized by spindled cells that invade following the direction of ECM fibers, vs. "amoeboid invasion," where rounded cells move between fibers in a less directional or more random manner [27,54,65]. Interestingly, tampering with mesenchymal invasion causes changes to invasive strategy (mesenchymal to amoeboid), yet fails to block invasion *in vivo *[25,26,66]. Knowing that MDA-MB-231 cells have undergone epithelial-to-mesenchymal transition [55], we questioned whether early or late stromal stages (e.g., control and tumor-associated 3D ECMs), could differentially regulate MDA-MB-231's behavior. What is more, it has been suggested that beta1-integrin dependent, yet PI3K independent pathways, could regulate ECM-induced Akt/PKB activity [67]. Therefore, we tested if interfering with pathways known to regulate cell invasion in general (e.g., Akt/PKB) or mesenchymal type of invasion in particular (beta1-integrin), would differentially affect the cells invading through early vs. late stromal ECMs. Figure 7 summarizes our results depicting all the cell trajectory tracks obtained for each condition tested (for representative videos see Additional files 4, 5 and 6; Movies 4–6). The relative spread of the star-like graphs in this figure, depicts the cell velocities where bigger stars represent fast cell movements. By looking at the patterns of the tracks, it is evident that conditions sustaining directional movements presented relatively straight line-tracks while wiggle lines represented directions that are more random. In regards to relative track orientations, the agglomeration of tracks near the X-axis shows the degree of tracks that were found to orient towards the most common angle on each experimental condition. Therefore, as a synopsis, we observed that indeed both early (control) and late (tumor-associated) 3D ECMs support a mesenchymal type of invasive behavior in MDA-MB-231 cells by inducing beta1-integrin regulated spindled morphology (Figure 3) and a relatively fast (Figure 4) and directional migration (Figure 5). Inhibition of beta-1 integrin effectively blocked the mesenchymal type of invasion supported by early matrix. Nevertheless, in late matrices, beta-1 integrin inhibition prompted little effect in velocity, while cellular morphology, directionality, and relative track organization of the trajectories were greatly influenced. This data suggests that, in the late-stage matrix, beta-1 integrin inhibition induces a change of invasive strategy. As a result, we believe that it is possible that tumor-associated (late), but not control (early), 3D ECMs support alternative, other than mesenchymal type of MDA-MB-231, invasion. Whether this altered invasive behavior is "amoeboid-like," is out of the scope of this work yet, the fact that the invasive behavior is random (as opposed to directional) and the cells present active blebs only in late 3D matrices under beta1-integrin inhibition (compare Additional file 4 and 5 (Movies 4 and 5)), strongly support this idea. We observed that beta1-integrin and/or PI3K pathways differentially regulate early vs. late stromal matrix induced (*in vivo*-like) invasive behavior. Furthermore, this work supports the notion that cells can behave differently within early vs. late stromal ECMs, and that both "cells" and "matrices" need predisposition to attain favorable invasion. For example, inhibition of PI3K or, to a better extent, blocking beta1-integrin function could potentially reduce invasive cell velocities in early (control) 3D matrices, yet combinations of these drugs would be counteractive. On the other hand, in late stage stromal matrices (tumor-associated), this combinatorial approach inhibits velocities to the same extent as beta1-integrin blockage alone. Perhaps, beta1-integrin inhibition in combination with that of additional pathways will prove to be more effective in the future for inhibition of possible alternative invasive strategies.

## Conclusion

Our data suggests that while both early and later matrices sustain mesenchymal invasion of MDA-MB-231 cells, only late stromal 3D matrices support a change of invasive strategy triggered by beta1-integrin inhibition. Therefore, our observations imply that "staging" stromal matrices, analogously to clinically relevant "tumor staging," might be an important step in assertively selecting invasive drug inhibitors. In addition, this work presents novel matrix-based assays to score tumor cell invasiveness and stroma permissiveness. These assays can be performed in a relatively short period of time, so that the stage of matrices produced *in vitro *accurately mimic the *in vivo *tumor microenvironment of the patient. It is possible that future applications of these assays could be used to score the effectiveness of targeted cancer drugs in inhibiting different aspects of cancer cell metastasis. Hence, this study could one day facilitate the identification of individuals at increased risk of recurrence, which remains a considerable challenge in the field [68], as well as personalized effective treatments.

## Competing interests

The authors declare that they have no competing interests.

## Authors' contributions

RCC, as first author, made substantial contributions to the conception and design of the manuscript, and either acquired or supervised the acquisition of all data in addition to assisting in the analysis and the interpretation of the data. DRK was instrumental in the Western blot analyses, as well as being an avid participant in the discussions that lead to the general conception of the paper. JS, acquired much of the invasion data and was very instrumental in the development of tools used to analyze the time-lapse data. MV, assisted in many of the experiments and was also instrumental in the discussions that lead to the final version of the manuscript. EC, drafted the manuscript and was instrumental in its design in addition to conceiving and directing the project. All authors were involved in revising the manuscript and all have given final approval of the version to be published.

## Pre-publication history

The pre-publication history for this paper can be accessed here:

http://www.biomedcentral.com/1471-2407/9/94/prepub

## Supplementary Material

Additional file 1 **Normal cell motility through staged 3D ECMs.** Montage of six hour time-lapse videos depicting MCF-10A cells moving through control (top left) and tumor-associated (top right) 3D ECMs or on 2D (bottom left). Click here for file

Additional file 2 **Tumorigenic cell motility through staged 3D ECMs.** Montage of six hour time-lapse videos depicting MCF-7 cells moving through control (top left) and tumor-associated (top right) 3D ECMs or on 2D (bottom left). Click here for file

Additional file 3 **Invasive cell motility through staged 3D ECMs.** Montage of six hour time-lapse videos depicting MDA-MB-231 cells moving through control (top left) and tumor-associated (top right) 3D ECMs or on 2D (bottom left). Click here for file

Additional file 4 **Invasive cell motility through control 3D ECMs under PI3K and/or beta-1 integrin inhibition.** Montage of six hour time-lapse videos depicting MDA-MB-231 cells invading through control 3D ECMs (top left) in the presence of 10 nM Wortmannin (top right), 50 μg/ml mAb13 (bottom left) or a combination of both mAb13 and 10 nM Wortmannin (bottom right). Click here for file

Additional file 5 **Invasive cell motility through tumor-associated 3D ECMs under PI3K and/or beta-1 integrin inhibition.** Montage of six hour time-lapse videos depicting MDA-MB-231 cells invading through tumor-associated 3D ECMs (top left) in the presence of 10 nM Wortmannin (top right), 50 μg/ml mAb13 (bottom left) or a combination of both mAb13 and 10 nM Wortmannin (bottom right). Click here for file

Additional file 6 **Invasive cell motility through staged 3D ECMs under PI3K and/or beta-1 integrin inhibition.** Montage of six hour time-lapse videos depicting MDA-MB-231 cells within 3D control (bottom panels) or tumor-associated (top panels) matrices, in the presence of 50 nM Wortmannin alone (left panels) or in combination with 50 μg/ml mAb13 (right panels). Click here for file
